# Nickel-Induced Reduced Graphene Oxide Nanoribbon Formation on Highly Ordered Pyrolytic Graphite for Electronic and Magnetic Applications

**DOI:** 10.1021/acsanm.3c05949

**Published:** 2024-05-11

**Authors:** Maximina Luis-Sunga, Alejandro González-Orive, Juan Carlos Calderón, Ilaria Gamba, Airán Ródenas, Teresa de Los Arcos, Alberto Hernández-Creus, Guido Grundmeier, Elena Pastor, Gonzalo García

**Affiliations:** † Instituto Universitario de Materiales y Nanotecnología, Departamento de Química, Universidad de La Laguna (ULL), PO Box 456, La Laguna, Santa Cruz de Tenerife 38200, España; ‡ Department of Technical and Macromolecular Chemistry, Paderborn University, Warburger Str. 100, Paderborn 33098, Germany; § Departamento de Física, Facultad de ciencias, Universidad de La Laguna, Avda. Astrofísico Francisco Sánchez, S/N, La Laguna, Santa Cruz de Tenerife 38200, Spain; ∥ Instituto Universitario de Estudios Avanzados (IUdEA), Departamento de Física, Universidad de La Laguna, PO Box 456, La Laguna, Santa Cruz de Tenerife 38200, España

**Keywords:** nickel, reduced graphene oxide, nanoribbons, 1D, self-assembly, nanomaterials, superparamagnetism

## Abstract

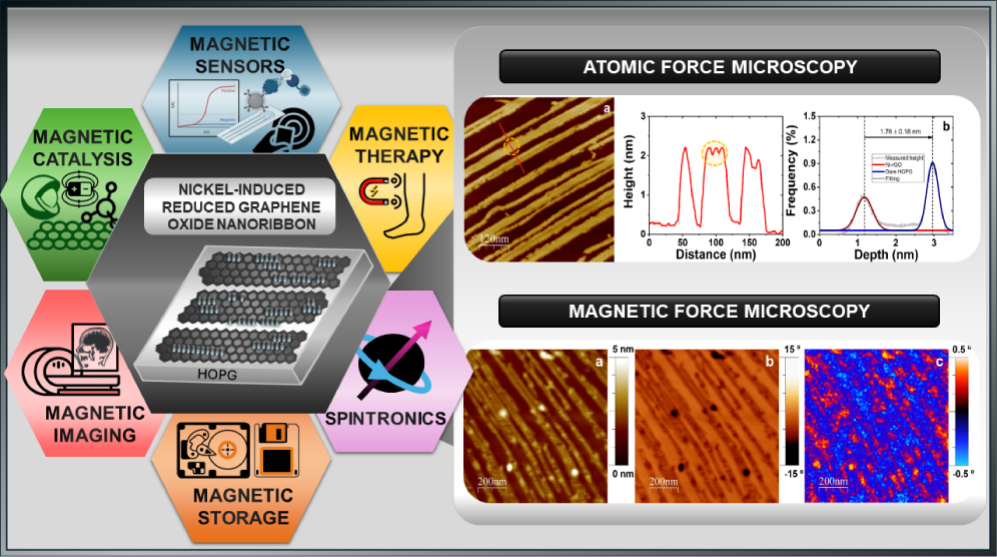

The development of nanoribbon-like structures is an effective strategy to harness the potential benefits of graphenic materials due to their excellent electrical properties, advantageous edge sites, rapid electron transport, and large specific area. Herein, parallel and connected magnetic nanostructured nanoribbons are obtained through the synthesis of reduced graphene oxide (rGO) using NiCl_2_ as a precursor with potential applications in nascent electronic and magnetic devices. Several analytical techniques have been used for the thorough characterization of the modified surfaces. Atomic force microscopy (AFM) shows the characteristic topographical features of the nanoribbons. While X-ray photoelectron spectroscopy (XPS), X-ray diffraction (XRD), and Raman spectroscopy provided information on the chemical state of Ni and graphene-like structures, magnetic force microscopy (MFM) and scanning Kelvin probe microscopy (SKPFM) confirmed the preferential concentration of Ni onto rGO nanoribbons. These results indicate that the synthesized material shows 1D ordering of nickel nanoparticles (NiNPs)-decorating tiny rGO flakes into thin threads and the subsequent 2D arrangement of the latter into parallel ribbons following the topography of the HOPG basal plane.

## 1 Introduction

Hybrid structures based on low-dimensional materials have received special interest due to the synergistic effects generated by integrating distinctive components at the molecular level, which can increase the range of application areas including environmental, energy and electronics.^1^ The investigation of two-dimensional (2D), one-dimensional (1D), and zero-dimensional (0D) materials has increased in the last years.^2^ Nanomaterials are considered to be materials smaller than 100 nm in at least one of their dimensions. Depending on the number of dimensions below 100 nm, we can find materials classified between 0 and 3 dimensions. 0D materials (quantum dots, nanoparticles) have their three dimensions below 100 nm, 1D materials (nanotubes, nanorods) possess two nanometric dimensions below 100 nm, 2D materials (graphene sheets) have 1 dimension below 100 nm, and 3D materials do not possess any dimension smaller than 100 nm (nanocomposites and nanomaterials-based dispersions).^3^ The electronic structure of this type of material differs from the same in the bulk, and the quantum confinement of the electrons can provide attractive and unique characteristics. In addition, these materials have a large chemically active surface area with great potential in a wide variety of sectors, including energy storage, catalysis, solar cells, and sensors. Therefore, investigating these materials can open paths with very promising results for a wide range of devices.^4^

Two-dimensional graphene oxide gained attention in the last years due to its high surface area, flexibility, and large number of oxygenated functional groups. In this type of material, the group of graphene nanoribbons reveals a structure that lies between 1D carbon nanotubes and 2D graphene sheets. They exhibit high chemical stability, high mechanical resistance, and excellent thermal and electrical conductivities. The band gap of nanoribbons changes with the width of the ribbon, and a finite band gap is obtained when the width is less than 10 nm. In addition, they exhibit high electron/hole mobility, photoconductivity, fast photocurrent response, and high sensitivity for sensor applications,^5^ besides its high surface area that increases the number of active sites on the surface.

The size or width of the nanoribbons significantly affects their electrical and magnetic properties, and these parameters can be modified to influence their optical characteristics. Indeed, large surface area of the nanoribbons favors a stronger interfacial interaction by increasing the number of active edge sites, leading to beneficial properties in fields such as batteries, solar cells, supercapacitors, and catalysis.^6,7^ In addition, their excellent flexible properties allow their application for portable devices, and their tuning with doping, defects/vacancies engineering, edge functionalization, or formation of heterostructures promotes the alteration of their optoelectronic characteristics, in order to be applied in different devices.^6−9^ The unique combination of magnetic properties and the specific structure of nanoribbons opens up possibilities for innovative applications in electronics. The ability to manipulate magnetism offers significant opportunities, from improving efficiency in advanced magnetic sensors to exploring the emerging field of spin electronics. In the medical field, nanoribbons offer potential for controlled drug delivery and improvements in magnetic imaging.^10^ In magnetic catalysis, they can act as catalysts, facilitating specific chemical reactions, and display capability in the fabrication of advanced magnetic sensors.^5,11^ In magnetic information storage, the improved properties of nanoribbons contribute to more efficient systems.^12^ In addition, they have significant potential in spin electronics, promoting advances in spintronics devices.^13^ Furthermore, their application in emerging energy technologies, taking advantage of their magnetic properties, is emerging as a promising area of research.^4^ It is essential to note that these applications represent developing areas of study, and the presence of enhanced magnetic properties opens opportunities to improve the functionality of electronic components by manipulating and controlling magnetism.

Regarding the change of these materials, graphene materials have been widely modified by adding nonmetallic heteroatoms (N, P, S) and transition metals.^9,14^ In particular, graphene nanoribbons have been altered with nitrogen to study their magnetic and electronic properties.^15,16^ Cortizo-Lacalle and coworkers built N-doped graphene nanoribbons with different lengths by means of an iterative coupling of dibenzodiazatetracene cores functionalized with *o*-diamines and diacetal-protected *o*-dione.^17^ The electronic and magnetic properties previously determined by theoretical calculations were verified from the physicochemical, electrochemical, and HOMO–LUMO characterizations, stating them as potential materials for field-effect transistors, photodetectors, solar cells, and molecular wires.^17^

On the other hand, transition metal doping can regulate the electronic properties (between metallic, semimetallic, and semiconductor) and the magnetic moment (among 0–5 μB) of graphene-based materials.^18^ The adjustment of these properties can provide valuable information to modify the catalytic activity and to obtain profitable catalysts for the design of different electronic devices.^19^ Jaiswal et al. performed a research based on spin-unrestricted density functional theory DFT calculations to compare the stability between Ni-terminated and one edge at Ni-doped armchair graphene nanoribbons.^20^ The authors found that one edge Ni-terminated graphene nanoribbons possess more stable energetic conditions than undoped graphene, a fact that was attributed to the stronger binding force between Ni and graphene and the coordination number of Ni.^20^ Furthermore, thermal properties of Ni-modified graphene nanoribbons suggest that the size of the ribbons influences the thermal conductivity of the materials, especially in the case of the Ni-coated graphene nanoribbons.^21^

Despite these studies, no abundant reports exist about the synthesis and characterization of Ni-doped or Ni-modified graphene nanoribbons and their potential as materials for the manufacture of electronic devices. It is also clear that no studies consider the effects of the formation of well-organized nickel nanoparticles supported on graphene nanoribbons, which could have special and useful magnetic, SERS-enhanced, and sensor properties.^22,23^

In this work, the preparation and physicochemical characterization of parallel and connected superparamagnetic nanoribbon nanostructures are obtained through the synthesis of reduced graphene oxide (rGO) using NiCl_2_ as a precursor and supported on highly oriented pyrolytic graphite (HOPG) with the view of their potential application as magnetic materials for electronic devices.

## 2 Materials and Methods

Nickel nanoparticles (NiNPs) supported on reduced graphene oxide (rGO) were synthesized using the methodology adapted by Flórez-Montaño et al.^24^ Briefly, the metal loading was 20 wt % with respect to GO. In this synthesis, 0.15 g of graphene oxide (GO) and 0.15 g of NiCl_2_·6H_2_O (99%, Sigma-Aldrich) were dissolved in 5 mL of ultrapure water (Milli-Q water by Millipore). After that, 2 mL of 30% ammonium hydroxide (Sigma-Aldrich) was added under stirring. Then, 45 mL of ethylene glycol (Aldrich Reagent-Plus) was incorporated at room temperature. The mixture was stirred for several hours and subsequently heated on a hot plate set around 200 °C while stirring. The precipitate was washed once with ultrapure water and three times with acetone. Then, it was suspended in a crystallization dish and heated at 60 °C for drying overnight. To obtain Ni-rGO, the black precipitate was transferred to a ceramic crucible and heated at 450 °C in a tubular furnace (Carbolite, UK) under reducing gas atmosphere (5% H_2_, 95% N_2_ at 103.3 mL min^–1^, Air Liquide). The same procedure, but in the absence of NiCl_2_·6H_2_O, has been carried out for comparison purposes obtaining a typical size of rGO measuring 22.3 nm in diameter and 2.3 nm in thickness (see Figure S2).

Topographic atomic force microscopy (AFM) images were obtained in Peak-Force Tapping mode with a Dimension Icon microscope equipped with a Nanoscope V control unit (Bruker) operating in ambient air conditions at a scan rate of 1 Hz. To this end, ScanAsyst-Air-HR (130–160 kHz, 0.4–0.6 N·m^–1^, and nominal radius of 2 nm) tips, purchased from Bruker, were used. In order to minimize tip convolution effects affecting the NPs width, data obtained from AFM image profiling have been corrected according to Canet-Ferrer et al.^25^ For the characterization of both the shape and size of the nanoparticles and nanoribbons, NPs and NRs, respectively,^26^ freshly cleaved HOPG substrates were incubated for *t* = 1 h in a 20% (w/w) Ni-rGO-containing solution by drop casting a 100 μL droplet. After that, the modified surfaces were thoroughly rinsed with ultrapure water and dried under a N_2_ stream. For the statistical evaluation of the size and height of the NPs, cross-sectional profiling was carried out on isolated nanoparticles (NPs) from AFM images taken in at least three regions of two different, but equivalent, samples. The latter results were summarized in histograms.

Magnetic force microscopy (MFM) images were performed by interleaving the topographic scan (operating under soft tapping conditions) with the “lift mode” scan. Images were taken at a scanning rate of 1 Hz with tips coated with magnetic CoCr film (MESP, 1–5 N m^–1^) working at a drive frequency of 75 kHz.

The scanning Kelvin probe force microscopy (SKPFM) analysis was performed to determine the contact potential differences on the surface of the bare and modified substrates, according to the presence/absence of rGO ribbons and metallic nanoparticles. A Dimension Icon microscope equipped with a Nanoscope V control unit (Bruker) was used to perform the measurements in the amplitude-modulation (AM-KPFM) mode (two-pass technique). The microscope was operated inside an antinoise box equipped with an antivibration table. The measurements were carried out using Pt-coated cantilevers (SCM-PIT V2, Bruker, with a spring constant of 3.0 N/m and a nominal tip radius of 25 nm), in areas of 5.0 × 5.0 μm^2^, and setting the lift scan height to 50 nm. The images were obtained with a resolution of 512 × 512 lines, an amplitude of 0.5 V, and a scan rate of 0.5 Hz. No data flattening, smoothing, or inversion of the surface maps was carried out. The Volta potential images of rGO-modified HOPG show only positive values since the Pt tip is biased, which is indicative of a less noble carbon sp^2^ matrix; i.e., it exhibits a more anodic behavior than the Pt reference tip. Consequently, lower potential figures recorded in certain regions would mean cathodic character. Conversely, higher potentials would be indicative of enhanced anodic behavior. A patterned reference sample containing regular Al, Si, and Au domains (as purchased from Bruker) was measured with analogue Pt tips under the same experimental conditions used herein for the characterization of the rGO. The as-obtained SKPFM measurements are displayed in Figure S1. As expected, positive contact potential values were recorded at the Al regions of the reference sample. Only slightly positive (or even negative) values were registered in the case of Au regions. Intermediate positive potential figures were recorded for the Si domains. A much more detailed explanation on the nobility, polarity, referencing, and other precautions when working with SKPFM is provided in the seminal contributions reported by Su et al.^26^

The Raman spectra of the samples were obtained by an InVia Renishaw confocal Raman microprobe system (Renishaw RE 04plc, Gloucestershire, UK) equipped with a Leica DM 2500 M confocal microscope and an λ = 532 nm laser.

Diffraction patterns were recorded from 5° to 100° (2θ) using an incidence X-ray beam (λ = 0.15406 nm) emitted by Cu–K_α_ cathode at 40 kV and 20 mA in a PANalytical X’Pert-Pro diffractometer.

X-ray photoelectron spectroscopy was performed in a laboratory setup equipped with a μ-FOCUS 600 X-ray monochromator NAP source and a Phoibos Analyzer Phoibos 150 NAP Analyzer from SPECS. Details about the characteristics of the setup can be found in ref. (27). The μ-FOCUS source used monochromated AlK_α_ radiation at 1486.7 eV, and it was operated at 50 W, with a spot size of approximately 285 μm in diameter and a photon irradiance of 35 μW/mm^2^. The incidence angle of the X-rays is 56.5° with respect to the surface normal, and the measurements were done with a takeoff angle of 90°. The pressure at the measurement chamber was 10^–8^ mbar and at the detector stage was 10^–10^ mbar.

Survey spectra were taken with a pass energy of 100 eV. Core level spectra were recorded at a pass energy of 40 eV. No corrections to the binding energy scale are made in this work, and all spectra are shown as measured. Under these conditions, the Au 4f_7/2_ level of a sputtered clean gold sample was at 83.9 eV binding energy, with a fwhm of 0.93 eV.

The peak fitting shown in Figure S8 was done using the program Unifit.^28^ Either a Shirley-type (for the O 1s and c 1s peaks) or linear (for the Ni 2p_3/2_ peak) background was fitted together with the components. The peaks were fitted using a convolution of Gaussian and Lorentzian line shapes.

## 3 Results and Discussion

**Figure 1:**
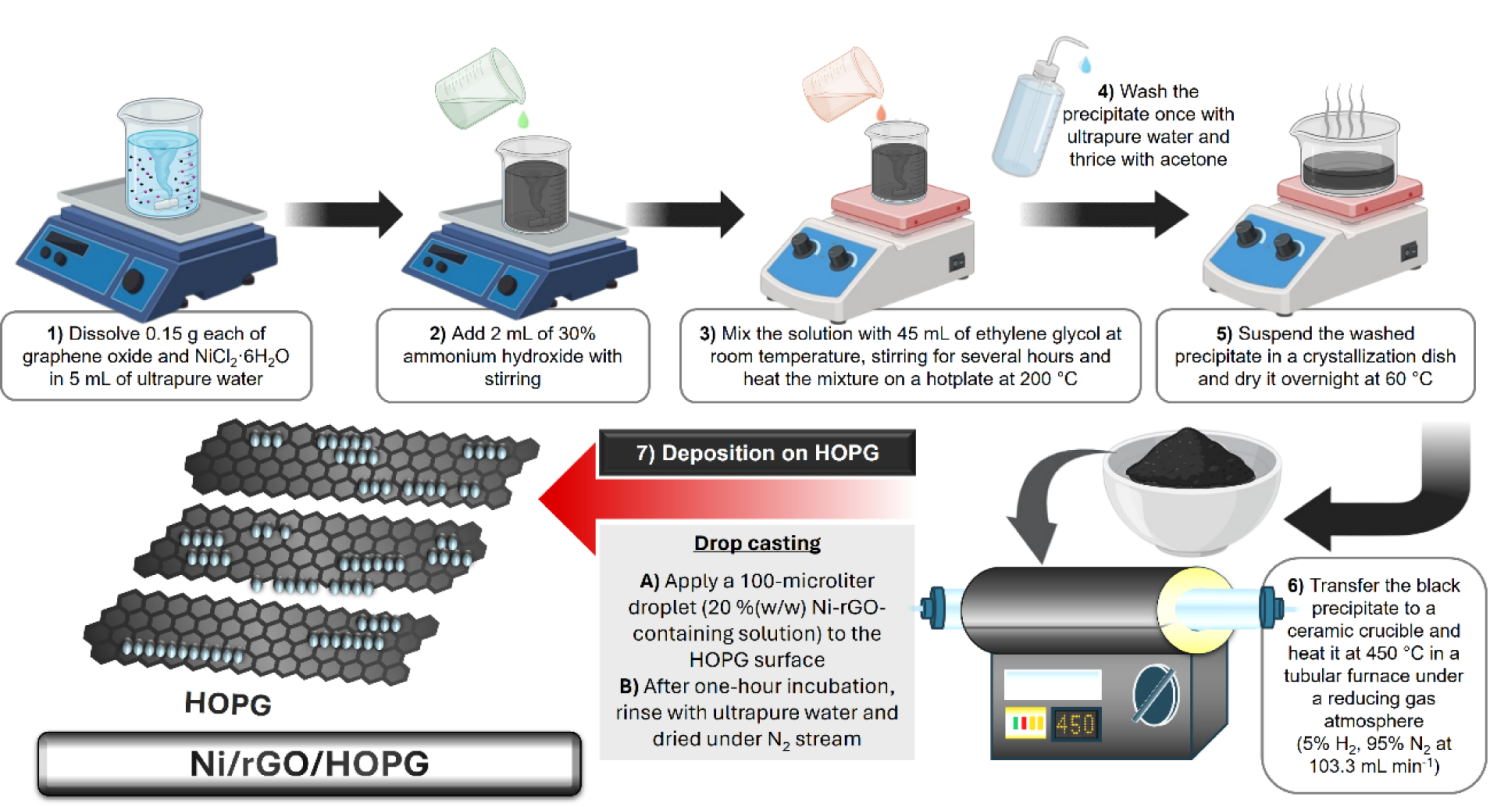
Schematic illustration of the step-by-step procedure followed for the synthesis of Ni-rGO nanoribbons on HOPG.

A graphical scheme that summarizes the key steps involved in the present work is listed in Figure 1.

The latter includes the following stages: preparation of Ni^2+^-GO particles involving steps 1–5 (i), followed by a heating treatment at 450 °C in a H_2_ reducing atmosphere intended to form Ni-rGO, step 6 (ii), and finally, the assembly of the Ni-rGO nanoribbons on the basal plane of the HOPG, step 7 (iii).

**Figure 2:**
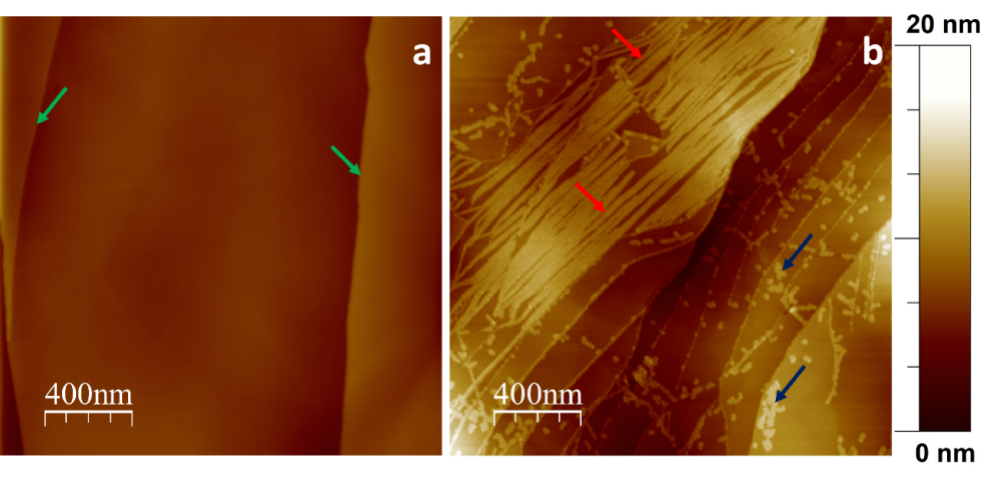
2.0 × 2.0 μm^2^ AFM images showing the characteristic topographic features of the basal plane of a freshly cleaved HOPG surface (a) and the 2D rGO nanoribbon self-assembly onto graphite terraces irradiated from HOPG steps (b).

AFM imaging was carried out in order to unveil the morphological features of the prepared Ni-rGO structures. The basal plane of a pristine HOPG surface exhibits the characteristic and featureless atomically flat terraces corresponding to sp^2^ hybridized carbon arranged in a hexagonal lattice limited by step edges identified with green arrows in Figure 2a. When NiCl_2_ is used as a precursor in the rGO synthesis process, the formation of nanostructures in the form of parallel and continuously connected nanoribbons can be observed in the AFM image depicted in Figure 2b. These structures would eventually 2D self-arrange aligned along a single crystallographic direction without intersecting each other. In addition to the ribbons (indicated by red arrows in Figure 2b), what may be identified as isolated tiny flakes (blue arrows) appear mostly decorating HOPG steps but also randomly distributed over the HOPG terraces. The size and height of these flakes are in very good agreement with those obtained from the preparation of rGO in the absence of NiCl_2_ and subsequent adsorption on HOPG, see Figure S2 for comparison. Interestingly, no nanoribbons were detected at all under these experimental conditions.

Nanoribbons in Figure 2 seem to be aligned forming 30, 60, 120, or 150° with the terrace step edges they irradiate from, indicating the preference to assemble following the topography of the underlying HOPG (0001) basal plane.^29^ This can be better appreciated in Figure S3. Indeed, Koç et al. showed by AFM imaging the existence of linear structures of ordered self-assembled SiO_2_ nanoparticles on the basal plane of the HOPG, which were also deposited by drop casting and seem to follow HOPG Moiré superlattices,^30,31^ i.e., which are known to reproduce the honeycomb structure of the HOPG basal plane and lead to the assembly of organic molecules into stripe-like shaped motifs.^32^ This would confirm that the formation of such striped structures, templated by Moiré pattern, is a growth model that is promoted during the dewetting process of a drop-casted colloidal solution.

**Figure 3:**
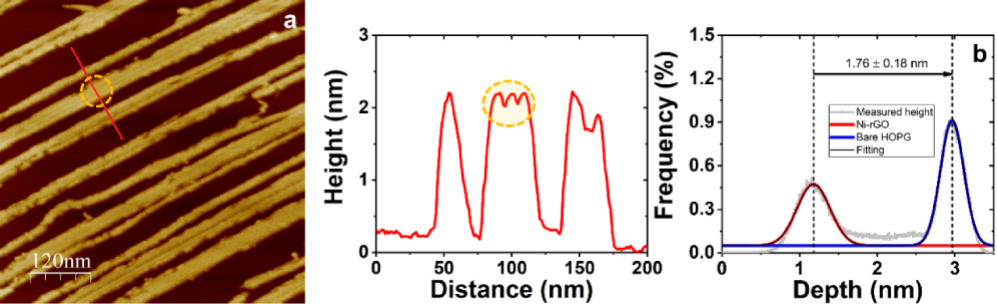
600 × 600 nm^2^ AFM image and representative cross-sectional profile showing the dimensions of nanoribbons (a). Dashed yellow circles are included as a guide for the eye to indicate that nanoribbons are formed by the 2D assembly of individual threads. A depth profile histogram exhibiting the height value distributions related to bare HOPG terrace, blue line, and Ni-rGO nanoribbons, red line, is displayed in (b). From the height difference between the two maximums of the Gaussian fits, the thickness of the Ni-rGO nanoribbons, i.e., 1.8 ± 0.2 nm, is obtained.

A closer look, see Figures 3a and FS4, allows us to conclude that nanoribbons are the result of the 2D assembly of individual threads, which turn out to be 5–8 nm wide. This value is noticeably lower than that registered for the width of rGO nanostructured tiny flakes deposited when the treatment was carried out in the absence of NiCl_2_ (22.3 ± 3.4 nm; see Figure S2). As can be deduced from the AFM images, the as-assembled nanoribbons are 20–50 nm wide, have 1.8 nm averaged height (as can be deduced from Figure 3b), and exhibit a considerable variation in length mostly dependent on the extension of the terrace considered.

**Figure 4:**
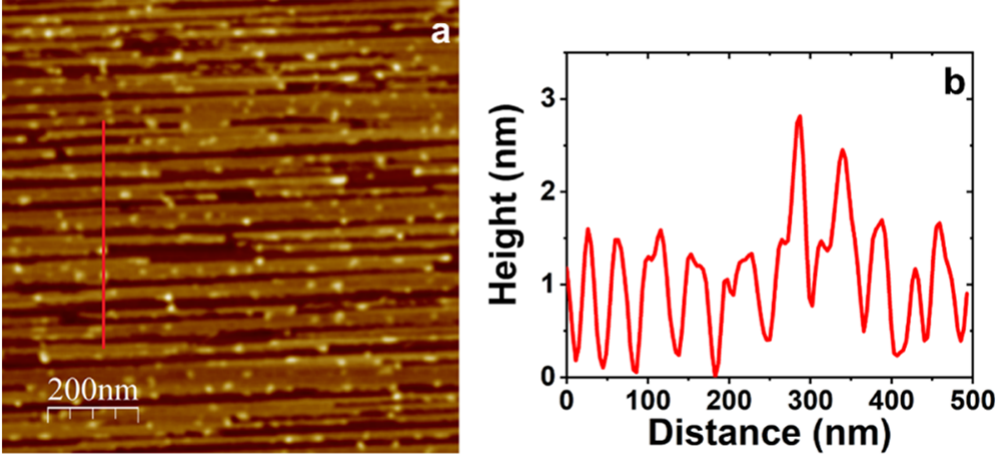
1.0 × 1.0 μm^2^ AFM image (a) and representative cross-section through the red line (b) showing in more detail that ribbons are decorated with bright rounded particles and their sizes and height, respectively.

Additionally, developing incomplete upper layers of rGO deposited over the ribbons can be observed in both the image and the cross-sectional profile (blue arrows in the image and highest peaks in the profile) of Figure S4. Defects and pinholes in the rGO matrix can also be distinguished in the image (dashed green circles). Indeed, as can be seen in Figure 4, the AFM images show that the ribbons are decorated with bright rounded nanoparticles (i.e., nanodisks) preferably located at these nanoribbon edges or defects. It is noteworthy that hydrogen/oxygen functional groups present at graphene edges and defects such as hydroxyl, aldehyde, and carboxylic groups are known to stabilize metal/metal oxide–graphene interfaces, promoting the trapping/adsorption of metal nanoparticles.^33,34^ The average value distribution of size and height collected from cross-section profiles carried out in AFM images taken at different regions and distinct but equally prepared, samples has been displayed as histograms in Figure S5.

Furthermore, the adhesion image displayed in Figure S6a shows different interfacial adhesion behavior related to the different physicochemical interactions occurring between them and the Si/SiO_2_ AFM tip.^35^ This trend can be better identified in Figure S6b, where the adhesion histogram accounts for three different, marked contributions, namely attributed to the basal plane of the HOPG, rGO ribbons, and nanodisks. Taking all these results into account, the latter may be attributed to the codeposition of Ni-based nanoparticles (NiNPs). The average size and height values are in very good agreement with those reported by Flórez-Montaño et al. for Ni@Pt nanoparticles adsorbed on rGO.^24^

**Figure 5:**
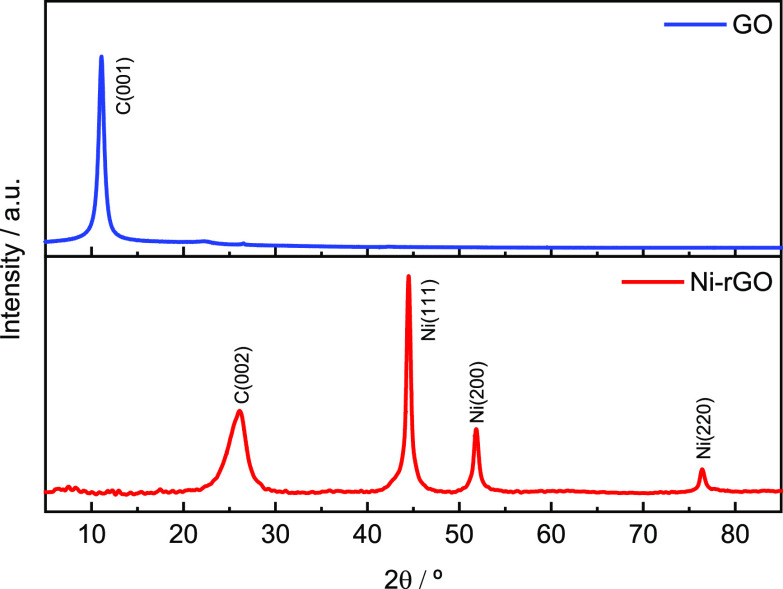
Diffraction patterns of GO (top) and Ni-rGO (bottom panel).

Figure 5 depicts X-ray diffractograms of GO and Ni-rGO powder materials. GO shows the typical contribution at low diffraction angles (11°) that is associated with the ⟨001⟩ diffraction plane.^36^ On the other hand, Ni-rGO material depicts four diffraction peaks. One peak at about 2θ = 26.0° that is from rGO and indicates the successful reduction procedure, and the peaks at 44.5, 52.0, and 76.5° can be assigned, respectively, to ⟨111⟩, ⟨200⟩, and ⟨220⟩ crystal planes of α-Ni(OH)_2_ (JCPDS no. 04-0850).^37^ It is further observed that NiNPs have a strong ⟨111⟩ orientation along the axial direction of the rGO nanoribbons, indicating a minimum specific free energy in this plane. Therefore, the material reveals typical XRD spectra of fcc-Ni structure with a single crystalline phase, but with a great intensity in the ⟨111⟩ orientation plane.

**Figure 6:**
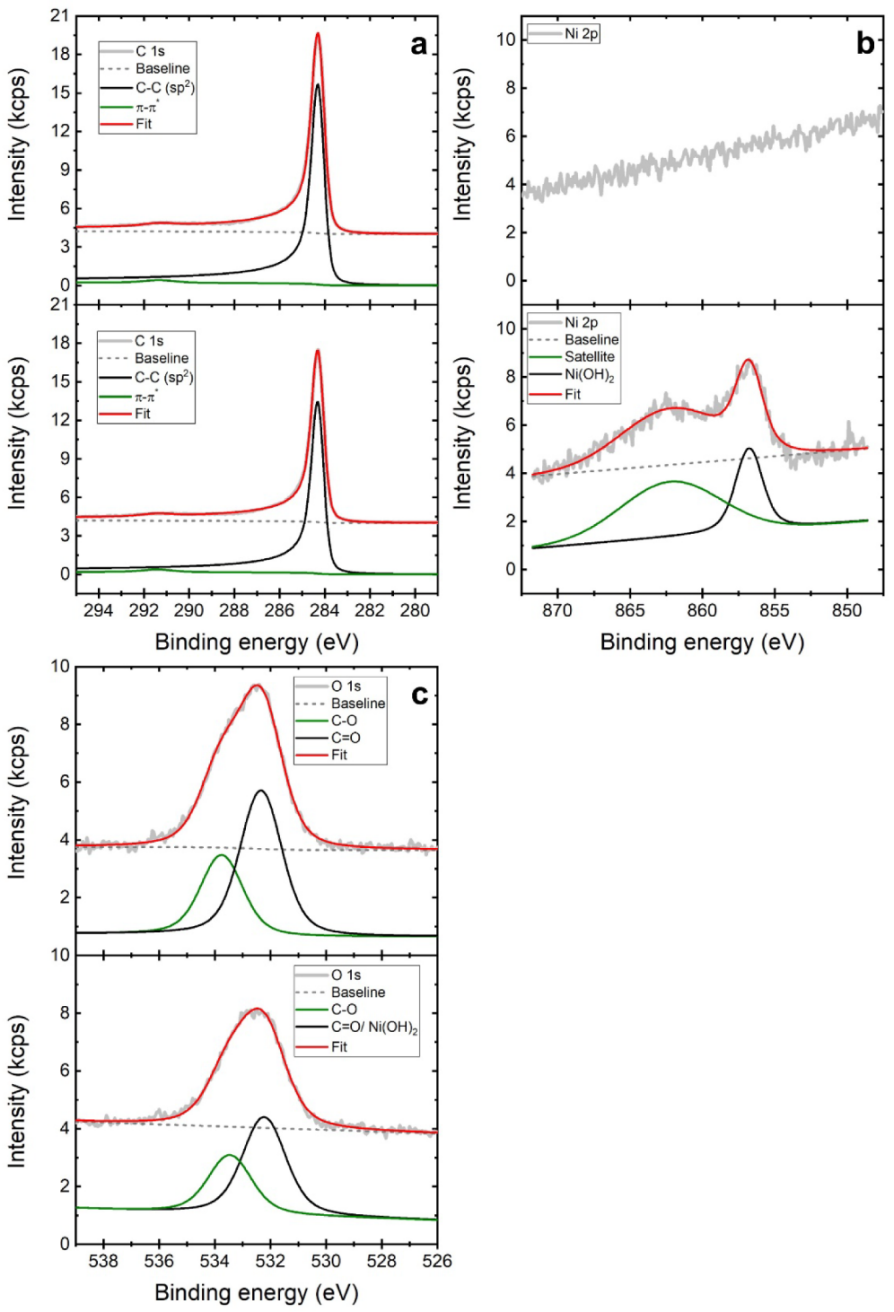
XPS core-levels of C 1s (a), Ni 2p (b), and O 1s (c) regions collected for the result of the deposition of rGO (top panels) and Ni-rGO (bottom panels) on HOPG.

The surface chemistry of rGO and Ni-rGO adsorbed on HOPG was characterized by means of XPS. C 1s core level XP spectra displayed in Figure 6a show feature characteristics of carbon crystalline structures based on C sp^2^ hybridization, i.e., the asymmetric contribution at 284.3 eV due to C–C sp^2^ and a π–π* shakeup peak at 291.4 eV.^38^ The latter is indicative of the successful reduction of GO to rGO upon heating at 450 °C in a H_2_-rich environment.

The Ni 2p_3/2_ core level spectrum displayed in Figure 6b exhibits two marked contributions at 856.8 and 862.3 eV, which may be assigned to Ni(II) in the form of Ni(OH)_2_ and a satellite peak, respectively.^39^ As expected, no traces of Ni were found in the rGO samples. The O 1s spectra shown in Figure 6c can be fitted to two contributions at 532.2 and 533.5 eV. While the first one may be assigned to oxygen doubly bounded to carbon (C=O), alcohols, and OH^–^ associated with metal in a solid oxyhydroxide phase such as Ni(OH)_2_,^40^ the latter would account for oxygen singly bound to carbon (C=O).^41^

**Figure 7:**
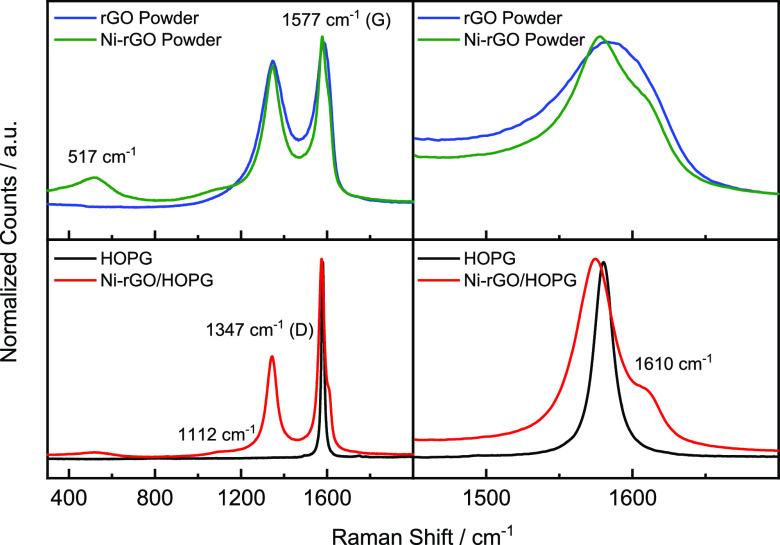
Raman spectra of rGO (blue line) and Ni-rGO (green line) powders (top panels) and Raman spectra of HOPG (black line) and Ni-rGO/HOPG (red line) materials (bottom panels).

Figure 7 depicts Raman spectra at the top panels for rGO (blue lines) and Ni-rGO (green lines) powders and at the bottom panels for HOPG (black lines) and Ni-rGO/HOPG (red lines) materials in which several contributions are clearly discriminated. The peaks at ca. 1347 cm^–1^ (D-band) and at ca. 1577 cm^–1^ (G-band) are originated from sp^3^ carbon domains and sp^2^ bonds into the graphitic grid, respectively.^42^ In the case of the Ni-rGO powder and Ni-rGO/HOPG materials, additional bands are observed at 517 cm^–1^ and at 1112 cm^–1^ that are attributed to acoustic lattice phonon and second-order lattice mode of α-Ni(OH)_2_.^43,44^ Furthermore, a small but visible contribution at ca. 1610 cm^–1^ broadening of the G-band at Ni-rGO powder suggests the activation of vibrational modes of adsorbed water as the responsible.^42^ Indeed, Ni-rGO/HOPG corroborates this supposition by the presence of a broad and big band at ca. 1610 cm^–1^ that is undoubtedly associated with the bending mode of adsorbed water (δ_OH_), which is strongly shifted to smaller Raman shift values than liquid-free water (Raman shift values are close to 1650 cm^–1^). Interestingly, the last indicates that any reaction in which water molecules are implicated will be strongly affected. It is also important to highlight that the presence of Ni species induced a narrowing of the full width at half-maximum (fwhm) at the powder and a displacement toward low Raman shift values of the G band at Ni-rGO powder and Ni-rGO/HOPG materials, which describes an increase in the order of the sp^2^ lattice.^44^ The last result is in good agreement with the formation of parallel nanoribbons observed by AFM.

**Figure 8:**
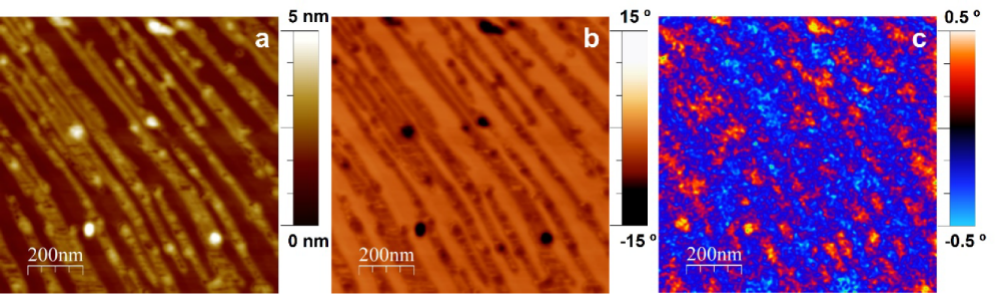
Typical 1.0 × 1.0 μm^2^ MFM images recorded for Ni-rGO adsorbed on HOPG. Topographical image (a), phase contrast image (b), and phase contrast image at a lift scan height of 50 nm (c). Blue and red regions would indicate different but parallel magnetization.

The magnetic properties of the ribbons have been assessed by means of MFM as can be seen in Figure 8. The topographic image in Figure 8a shows NiNPs decorating nanoribbons assembled on an HOPG terrace. In addition to Figure 8b, where the phase contrast image resembles the differential physicochemical properties of the HOPG basal plane, the rGO ribbons, and the NiNPs on top of the latter, while maintaining a clear correspondence with the topographic image, Figure 8c reveals the emergence of alternating positive (in red) and negative (blue) phase contrast domains. The latter is characteristic of the in-plane ferromagnetic coupling of superparamagnetic particles.^45−47^ The size of the magnetic particles is known to have a significant effect on their magnetic properties. While magnetic particles ranging from 30 to 80 nm are considered to behave like single-domain magnets, particles with diameters below that bottom threshold usually exhibit superparamagnetic behavior since their reduced size does not allow them to ensure a stable magnetic moment, which makes them vulnerable to thermal energy, which can reverse this magnetic moment.^48^ Similar ferromagnetic-like behavior to that here reported, but due to the 2D assembly of functionalized small magnetic nanoparticles, has been detected in the arrangement of 12 nm-sized oleic acid-coated Co nanoparticles.^49^ Additionally, although it has been reported that the presence of point defects in carbon sp^2^-based crystalline structures such as HOPG may be the origin of ferromagnetic ordering, these features were not observed herein for isolated rGO particles by MFM (data not shown).^50^

**Figure 9:**
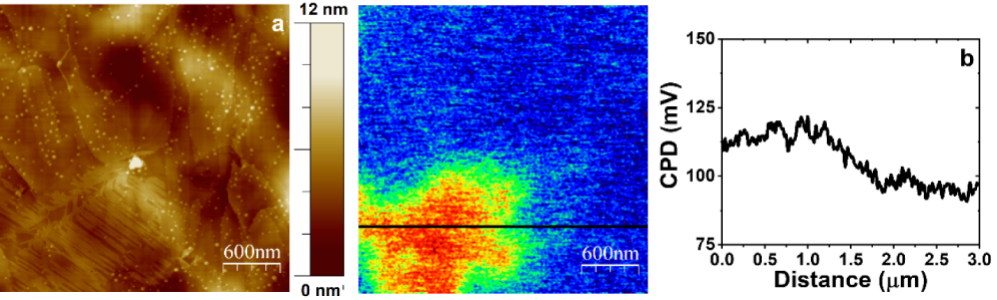
3.0 × 3.0 μm^2^ SKPFM image registered for Ni-rGO adsorbed on HOPG. Topographic image (a). CPD image and representative cross-sectional profile through the black line show the surface potential value distribution (b).

In this regard, the surface potential properties of the as-obtained structures were explored using SKPFM. Figure 9 shows the contact potential difference (CPD) value distribution associated with the different compositions of the structures adsorbed on HOPG terraces. While no significant variations can be observed in those regions where only isolated rGO tiny flakes could be observed (blue region in the surface potential map), a significant increase to more positive values of the CPD could be detected in those areas where nanoribbons are present (red region).

Since the Pt-AFM tip is biased in these experiments, more positive values are indicative of lower values of work function or more anodic-behavior materials.^51^ Interestingly, NiO, Ni(OH)_2_, and NiOOH are known to exhibit lower work function values than that corresponding to rGO and the basal plane of the HOPG.^52,53^ The latter would explain the more positive values in CPD observed for the nanoribbons (decorated with Ni nanoparticles) in comparison with those registered for bare HOPG terraces or small rGO flakes. These figures are in very good agreement with those of the tiny flakes produced using the same procedure, but in the absence of NiCl_2_, see Figure S7. MFM and SKPFM results put together hint at a significant concentration of Ni in the ribbons.

The affinity for and binding sites of Ni on graphene-based architecture still remain under debate.^54^ In this regard, some theoretical works have pointed out that while alkali metals attach to graphene structures by ionic bonding (with minor distortion in the graphene lattices), in the case of transition metals, they were expected to bind graphene by means of covalent bonds.^55^ However, experimental approaches have shown that transition metals would mostly adsorb onto graphene via ubiquitous hydrocarbon contamination instead of the free graphene surface.^56^ This would be related to a poor affinity between graphene and the transition metals. In addition, the presence of defects in rGO has been reported to be responsible for attaching transition metal nanoparticles.^57^

It can be proposed that during the heating treatment at 400 °C in H_2_/Ar atmosphere, Ni nanoparticles adsorbed at graphene defective sites catalyze carbon hydrogenation at reduced graphene edges tearing apart rGO tiny flakes (∼22 nm wide) into smaller building blocks.^58^ The latter would then adsorb by π-stacking and self-assemble, presumably starting by chemically binding to more active step edges and spreading then to the more inert terraces, into long threads ranging 5–8 nm wide resembling the crystalline features of the HOPG (0001) basal plane underneath, most likely due to the spontaneous 1D ordering driven by coupling of magnetic Ni nanoparticles.^59^ These threads would interact with each other by hydrogen bonding involving oxygenated functional groups located at the rGO edges and eventually packing into parallel nanoribbons. This fact opens the possibility to use these rGO nanoribbons as suitable components in the design of different electronic devices such as transistors and spintronics.^60^

## 4 Conclusion

In this work, the preparation and characterization of parallel magnetic Ni-modified rGO nanoribbons are reported. Modification of nanomaterials using approaches such as alloys, doping, defects, and heterostructure formation with nanoribbon-like structures can provide promising properties to device performance. The synthetic approach employed here is low cost, and the potential applications in the field of magnetic materials for electronic devices are enormous. This study contributes to the fundamental understanding of the properties of these nanoribbons and raises promising possibilities for technological advances in the design and fabrication of more efficient and versatile electronic devices. AFM imaging showed the formation of reduced graphene oxide (rGO) nanoribbons and tiny flakes obtained on the HOPG (0001) basal plane after its incubation in an aqueous solution containing the result of the reduction of graphene oxide by means of a heating treatment with Ni precursors (NiCl_2_) in a reductive atmosphere. No ribbons were detected on the HOPG surface at all when the rGO is prepared upon the same experimental conditions but in the absence of NiCl_2_. The AFM images, together with the interfacial adhesion analysis, allowed us to conclude the occurrence of the codeposition of Ni nanoparticles, which mostly appeared decorating the rGO nanoribbons. While XRD, XPS, and Raman analyses confirmed that Ni nanoparticles are formed by Ni(OH)_2_, and the successful reduction of GO to rGO, MFM, and SKPFM results showed a significant concentration of Ni in the ribbon-rich regions. The presence of defects and edges on rGO nanoribbons is proposed to be responsible for the attachment of the Ni nanoparticles. The mechanism for the nanoribbon formation involves tiny graphene flakes bearing NiNPs adsorbing by π-stacking and self-assemble into long threads following the crystalline features of the HOPG (0001) basal plane terraces underneath, which is most likely assisted by the magnetic coupling of NiNPs. The as-formed threads would interact with each other by hydrogen bonding to eventually pack into parallel nanoribbons. This contribution may serve as a proof of concept that demonstrates that using NiCl_2_ as a precursor induces significant changes in the rGO self-assembling properties on HOPG. More studies are needed in order to gain a deeper understanding of the effect that the amount of Ni may have on nanoribbon size and are currently underway in our laboratory. Such experiences are of fundamental importance for future applications concerning the control of the NiNPs-rGO composite shapes and the assembly of features using external localized magnetic fields. To conclude, the synthesis of parallel magnetic Ni-based nanoribbons opens exciting prospects in the field of electronics and magnetics, thanks to the possibility of modulating their magnetic properties. This lays a solid foundation for future research and development at the intersection of nanotechnology and magnetic electronics.

## Supporting Information Available

SKPFM images of an Al–Si–Au patterned grid (Figure S1); 2.0 × 2.0 μm^2^ AFM image and representative cross-section through the red line showing in more detail small, rGO tiny flakes supported on freshly cleaved HOPG (Figure S2); 600 × 600 nm^2^ AFM image showing 2D Ni-rGO nanoribbon self-assembly onto graphite terraces (Figure S3); 600 × 600 nm^2^ AFM image and representative cross-section through the red line, showing in more detail that ribbons are formed by the 2D self-assembly of parallel threads (Figure S4); histograms showing averaged value distributions of size and height (Figure S5); 1.0 × 1.0 μm^2^ adhesion AFM image and representative cross-section through the red line showing in more detail that ribbons are decorated with dark rounded Ni nanoparticles (Figure S6); and 3.0 × 3.0 μm^2^ SKPFM image registered for rGO adsorbed on HOPG (Figure S7) (PDF)
