# Supplementary material for: Nickel-Induced Reduced Graphene Oxide Nanoribbon Formation on Highly Ordered Pyrolytic Graphite for Electronic and Magnetic Applications
Source: ACS Appl Nano Mater. 2024 May 11;7(10):11088–96. doi: 10.1021/acsanm.3c05949 (PMC11131383; doi:10.1021/acsanm.3c05949)
Supplement: Supplementary file 1 — an3c05949_si_001.pdf [file an3c05949_si_001.pdf]

Supporting Information

Nickel-Induced Reduced-Graphene Oxide Nanoribbon Formation on Highly Ordered Pyrolytic Graphite for Electronic and Magnetic Applications

Maximina Luis Sunga[1], Alejandro González Orive[1,2], Juan Carlos Calderón Gómez[1,2], Ilaria Gamba[1], Airán Ródenas[3,4], Teresa de los Arcos[2], Alberto Hernández Creus[1], Guido Grundmeier[2], Elena Pastor[1], Gonzalo García[1,*]

[1]*Instituto Universitario de Materiales y Nanotecnología, Departamento de Química, Universidad de La Laguna, PO Box 456, 38200, La Laguna, Santa Cruz de Tenerife, España.*

[2]*Department of Technical and Macromolecular Chemistry, Paderborn University, Warburger Str. 100, 33098 Paderborn, Germany.*

[3]*Department of Physics, Universidad de La Laguna, Avda. Astrofísico Francisco Sánchez, S/N, Facultad de ciencias, La Laguna, Santa Cruz de Tenerife 38200, Spain*

[4]*Instituto Universitario de Estudios Avanzados (IUdEA), Departamento de Física, Universidad de La Laguna, PO Box 456, 38200, La Laguna, Santa Cruz de Tenerife, España.*

**Corresponding author: ggarcia@ull.edu.es*

Table of contents

SKPFM images of an Al-Si-Au patterned grid……………………………………………………………S-2

AFM image of rGO tiny flakes supported on freshly cleaved HOPG………………………………S-3

AFM image showing 2D Ni-rGO nanoribbon self-assembly onto graphite terraces……………………………………………………………………………………S-4

AFM image of ribbons formed by the 2D self-assembly of parallel threads……………………………………………………………………………………S-5

Histograms distributions of size and height of brightest rounded nanoparticles present on top of the nanoribbons……………………………………………………………………………S-6

Adhesion AFM image of ribbons decorated with dark rounded Ni nanoparticles………………………………………………………………………………S-7

SKPFM image registered for rGO adsorbed on HOPG……………………………………………S-7

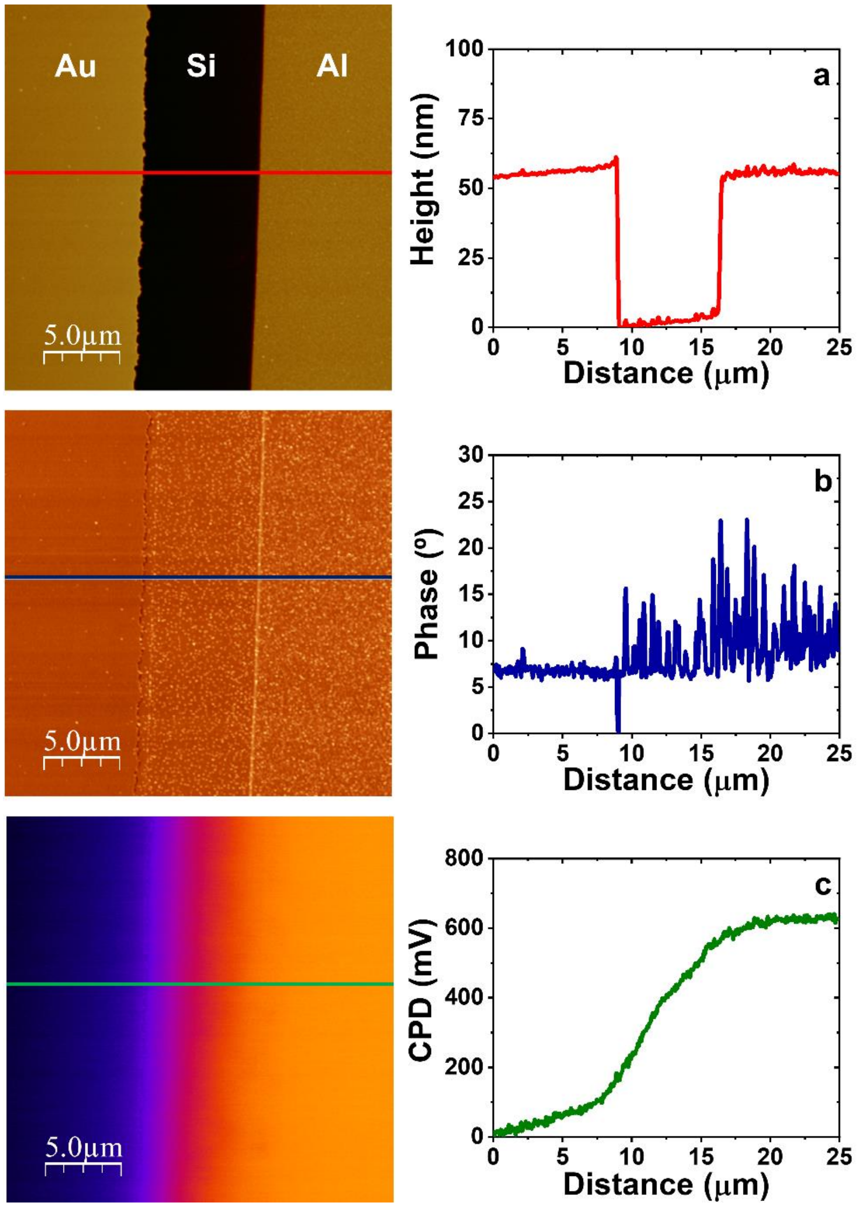

Figure S1. SKPFM images of an Al-Si-Au patterned grid (collected when the Pt-coated tip is biased): Topographic (a), phase contrast (b), and contact potential difference (c) images. Right panels show the corresponding cross section profiles.

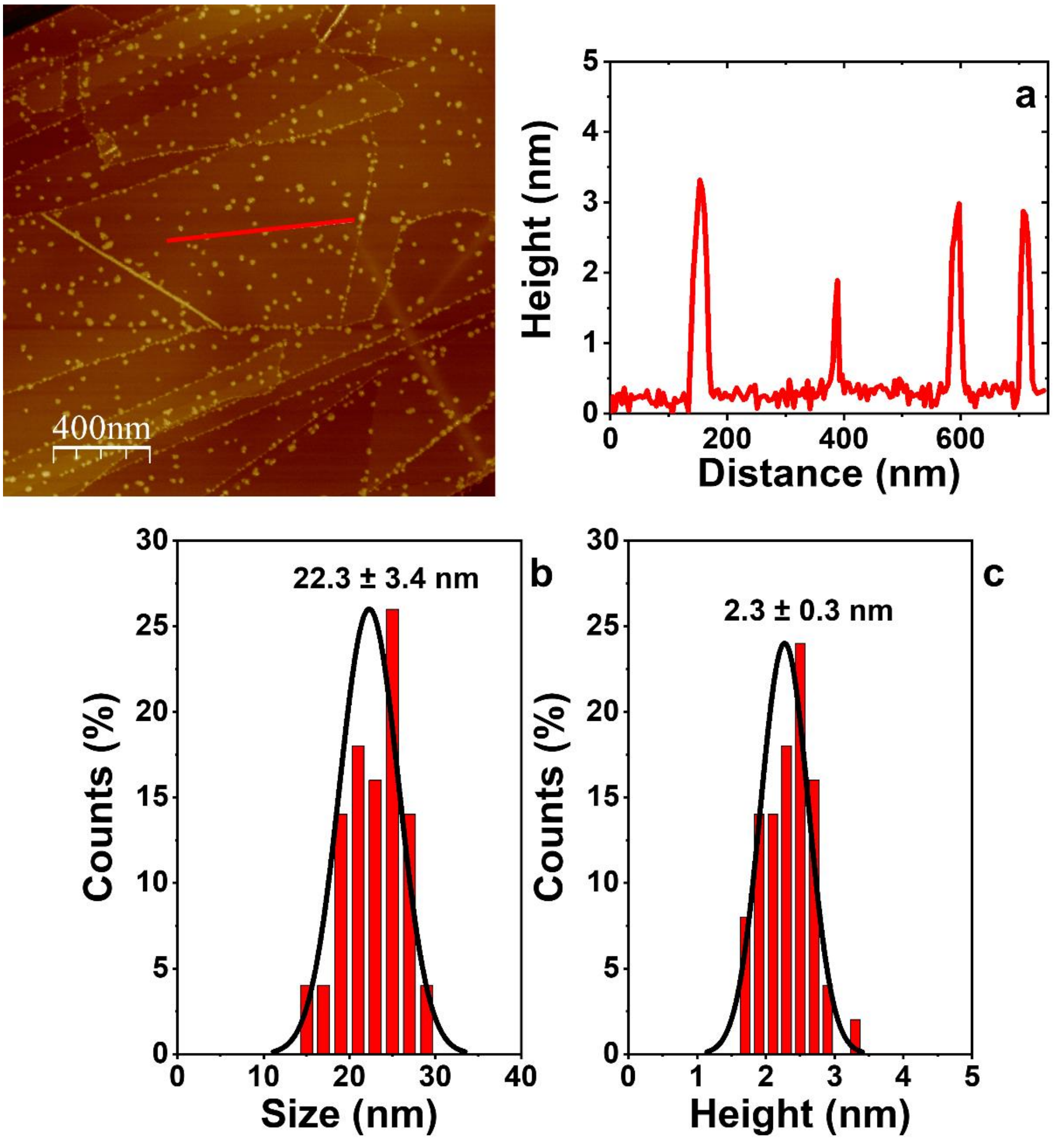

Figure S2. 2.0 x 2.0 µm^2 AFM image and representative cross section through the red line showing in more detail small, rGO tiny flakes supported on freshly cleaved HOPG (a). Histograms showing averaged value distributions of size (b) and height (c) of rGO obtained from cross section profiles taken in (a).

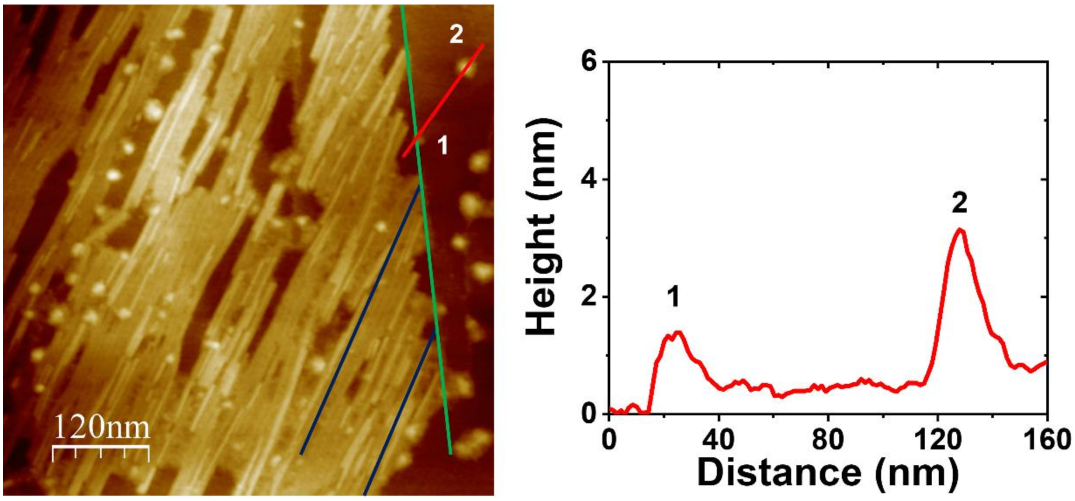

Figure S3. 600 x 600 nm^2 AFM image showing 2D Ni-rGO nanoribbon self-assembly onto graphite terraces which irradiates from HOPG steps (indicated in the image with a green line for clarifying purposes) forming $30/150^0$ (blue lines have been added to the image to guide the eye). A representative cross-section profile, right plot, has been carried out through the red line displayed in the AFM image in order to show the characteristic dimensions of isolated either Ni or rGO particles adsorbed on steps (1) or terraces (2).

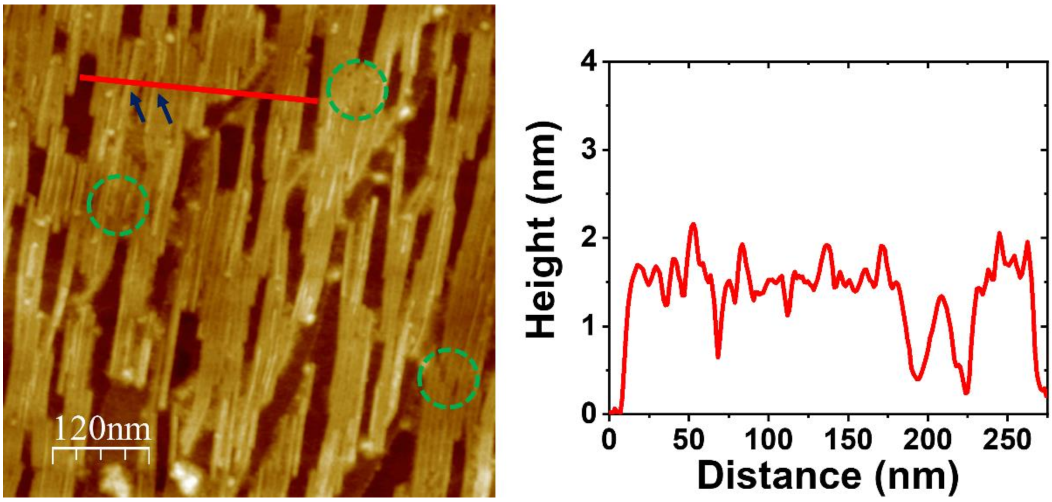

Figure S4. 600 x 600 nm^2 AFM image (left) and representative cross section through the red line (right) showing in more detail that ribbons are formed by the 2D self-assembly of parallel threads. Upper layers of rGO deposited over the ribbons can be observed in both the image and the cross-section profile (blue arrows in the image and highest peaks in the profile). Defects and pinholes in the rGO matrix can be distinguished in the image (dashed green circles), where isolated rounded bright Ni nanoparticles (or clusters of them) tend to be trapped (together with the edges of the debris or assembled ribbons).

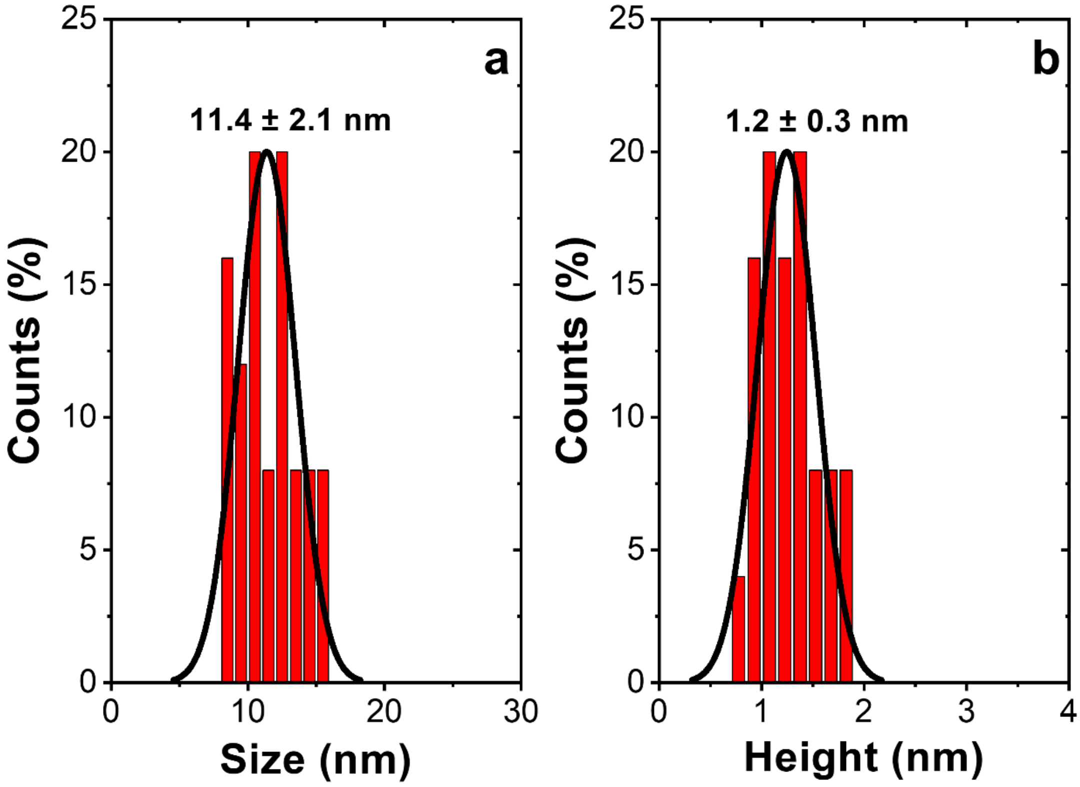

Figure S5. Histograms showing averaged value distributions of size (a) and height (b) have been obtained for the brightest rounded nanoparticles present on top of the nanoribbons and collected from representative cross section profiles taken in different AFM images.

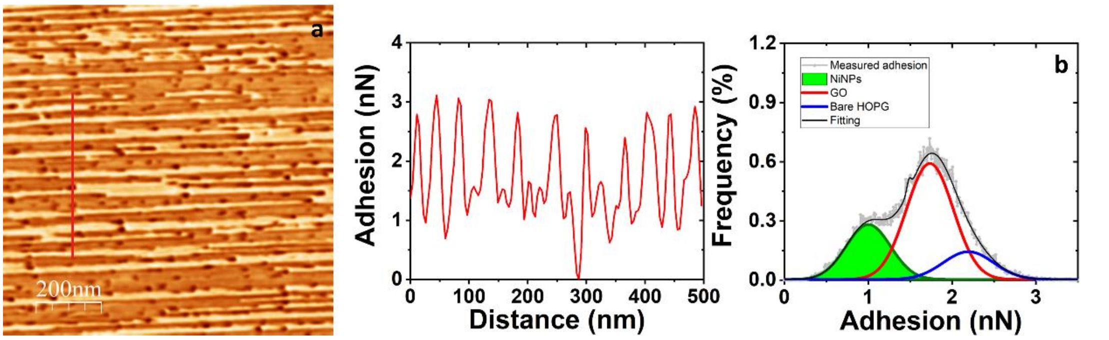

Figure S6. 1.0 x 1.0 μm^2 adhesion AFM image and representative cross section through the red line showing in more detail that ribbons are decorated with dark rounded Ni nanoparticles (a). Adhesion histogram showing three different contributions corresponding to bare HOPG terrace (blue line), rGO nanoribbons (red), and Ni nanoparticles (solid green) (b).

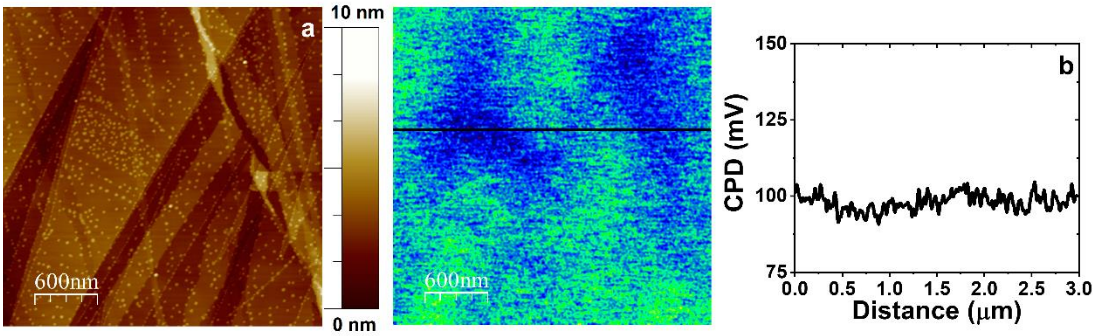

Figure S7. 3.0 x 3.0 μm^2 SKPFM image registered for rGO adsorbed on HOPG. Topographic image (a). CPD image and representative cross-sectional profile through the black line showing the surface potential value distribution (b).
